# Coexistence of craniopharyngioma and cranial fibrous dysplasia: a case series of clinicopathological study

**DOI:** 10.1186/s13023-022-02281-1

**Published:** 2022-03-18

**Authors:** Yang-Hua Fan, Zhi Li

**Affiliations:** Department of Neurosurgery, Beijing Tiantan Hospital, Capital Medical University, No.119, the South Fourth Ring West Road, Fengtai District, Beijing, 100070 China

**Keywords:** Craniopharyngioma, Cranial fibrous dysplasia, Coexistence, Clinicopathologic features, Treatment

## Abstract

**Background:**

Craniopharyngioma (CP) and cranial fibrous dysplasia (CFD) are rare embryonic benign cranial diseases that most commonly present during childhood or adolescence. The coexistence of CP and CFD is extremely rare and has not yet been reported.

**Methods:**

We retrospectively reviewed the data of five patients with concomitant CP and CFD treated at Beijing Tiantan Hospital from January 2003 to January 2021 and summarized their clinicopathological features, treatment modalities, and outcomes. We also performed a comprehensive literature review, tested the patients for characteristic *GNAS* gene mutations related to CFD, and tested the CP specimens for corresponding Gsα protein to explore the potential connection leading to the coexistence of CP and CFD.

**Results:**

The cohort comprised four men and one woman (median age, 39 years). The symptoms mainly included headache, dizziness, fatigue, polyuria/polydipsia, hypogonadism, and blurred vision. CFD most commonly involved the sphenoid bone (n = 4). Four patients underwent surgery to remove the CP (one trans-sphenoidal and three transcranial resections); complete and subtotal resection were achieved in two patients, respectively. The tumor subtype was adamantinomatous in three patients and unknown in one. The common postoperative complications were panhypopituitarism, diabetes insipidus, and hypothyroidism. The mean follow-up duration was 57.2 months. Two patients required postoperative hormone replacement therapy. Three patients underwent genetic study of the tumor specimens; *GNAS* mutations were not detected, but these patients were positive for Gsα protein.

**Conclusions:**

Although a definite causative relationship has not been proved, the coexistence of CP and CFD means that potential interplay or an atypical fibrous dysplasia course as uncommon manifestations of CP cannot be excluded. It is more challenging to initiate prompt diagnosis and appropriate treatment for concomitant CP and CFD than for solitary CP because of skull base deformations. Current management strategies are aimed at surgical treating the CP and regularly monitoring the CFD.

**Supplementary Information:**

The online version contains supplementary material available at 10.1186/s13023-022-02281-1.

## Introduction

Craniopharyngiomas (CPs) are rare epithelial tumors that arise along the path of the craniopharyngeal duct and account for 2%–5% of all primary intracranial neoplasms [1, 2]. The two major histological subtypes of CP are adamantinomatous CP (ACP) and papillary CP, and these subtypes differ in their genesis and age distribution [3, 4]. Although the histological grade of CP is usually low, patients with CP usually have significant morbidity and mortality rates, making it one of the most challenging to treat.

Fibrous dysplasia (FD) is a benign skeletal disorder caused by somatic *GNAS*-activating mutations [5]. FD accounts for 7% of benign bone tumors and most commonly affects the craniofacial regions [6, 7]. Patients may exhibit involvement of one bone (monostotic FD) or multiple bones (polyostotic FD) or they may have McCune-Albright syndrome (MAS), which is classically defined as the triad of polyostotic FD, café-au-lait skin macules, and endocrinopathies [8].

The disfigurement and thickening of cranial bone caused by coexistent cranial FD (CFD) makes the treatment of CP more complicated. The coexistence of CFD and CP has not yet been reported, and the pathogenesis of such coexistence remains unclear. In the present study, we present our experience with the successful treatment of five patients with concomitant CP and CFD, and discuss the optimal therapeutic strategy and potential links between these two lesions.

## Materials and methods

### Patients

We retrieved the medical records of all patients diagnosed with both CP and CFD in the Department of Neurosurgery, Beijing Tiantan Hospital, Capital Medical University from January 2003 to November 2021. A total of five patients were diagnosed with concomitant CP and CFD. Their demographic data, clinical manifestations, physical examination, surgery and treatment procedures, and outcomes were verified in the clinical medical reports. Patient follow-up data were obtained via outpatient and telephone follow-up. Endocrine data were measured by laboratory examination of hormone levels, and radiological data were obtained from the Picture Archiving and Communication System in our hospital. All patients underwent contrast-enhanced magnetic resonance (MR) imaging and computed tomography (CT) before and after treatment. This study was approved by the hospital ethics committee, and informed consent was obtained from all included patients at follow-up.

### Immunohistochemistry and genetic analysis

Immunohistochemistry was performed to detect Gsα protein expression in CP specimens. The tissue sections were incubated with primary Gsα antibody (1:100, sc-365855, Santa Cruz). Each stained slide was individually reviewed and independently scored by two neuropathologists.

Genomic DNA was extracted from paraffin-embedded CP specimens using the Wizard Genomic DNA Purification Kit in accordance with the manufacturer’s instructions (Promega, Madison, WI).

## Results

### Patient characteristics

The clinical, therapeutic, pathological, and outcome characteristics of the five patients are shown in Table 1. The study cohort comprised four men and one woman (median age, 39 years; range, 29–55 years). The mean follow-up time was 57.2 months (range, 6–98 months). The preoperative symptoms included headache (n = 2), dizziness (n = 2), polyuria/polydipsia (n = 2), fatigue (n = 2), visual deterioration (n = 1), and hypogonadism (n = 1). One patient (patient 5) was incidentally diagnosed with CP and CFD during a physical check-up. The mean duration of symptoms was 8.2 months (range, 1.0–24.0 months). None of the patients had received previous radiotherapy or surgery. The bone most commonly affected by FD was the sphenoid bone (n = 4), and none of the patients had previously been diagnosed with FD.

**Table 1.** Clinical, therapeutic, pathological and prognostic characteristics of the 5 patients included in this study

No.	Case 1	Case 2	Case 3	Case 4	Case 5
Gender/Age (year)	M/46	M/36	F/29	M/55	M/29
Onset symptoms	Dizziness,anorexia, polydipsia/polyuria, visual deterioration	Headache, poor energy, hypogonadism	Headache	Dizziness, poor energy, polydipsia/polyuria	Incidental
Disease duration (m)	24	12	1	3	1
Neurological findings (pre-op)	Visual acuity: L 0.12, R 0.4; bitemporal hemianopsia	NSPS	NSPS	cognitive/memory decline; weakness of lower limb	NSPS
Treatment and Approach	Transsylvian	Transcallosal	Transsphenoidal	(1) V-P shunt (2)Transsylvian	Watchful waiting
Extent of resection	STR	GTR	STR	GTR	Undone
Pathology	ACP	ACP	CP	ACP	Undone
GNAS mutation	Negative	Negative	Negative	Undone	Undone
GNAS expression	Positive	Positive	Positive	Undone	Undone
Endocrine deficits (pre-op)	Hypogonadism	Hypothyroidism; hyperprolactin	Hyperprolactin; hyperthyroidism	Hypothyroidism	Nomal
Endocrine deficits (post-op)	Panhypopituitarism	Hypothyroidism	Transient hypothyroidism	Panhypopituitarism	Nomal
Complications	Transient hypernatremia; central fever	Transient DI	Meningitis; visual loss	DI; thrombus of lower extremity veins	Undone
Follow-up time (m)	98	97	57	6	28
Outcome	Residual without progression; hormone replacement therapy	No recurrence	No recurrence	Died of pulmonary embolism	No progress

*M* Month, *Pre-op* Preoperative, *Post-op* postoperative, *DI* Diabetes insipidus, *NSPS* No significant positive symptoms, *ACP* Adamantinomatous craniopharyngioma, *PCP* Papillary craniopharyngioma, *STR* Subtotal resection, *GTR* Gross-total resection, *V-P* Ventriculo-peritoneal

### Radiographic characteristics

**Fig. 1:**
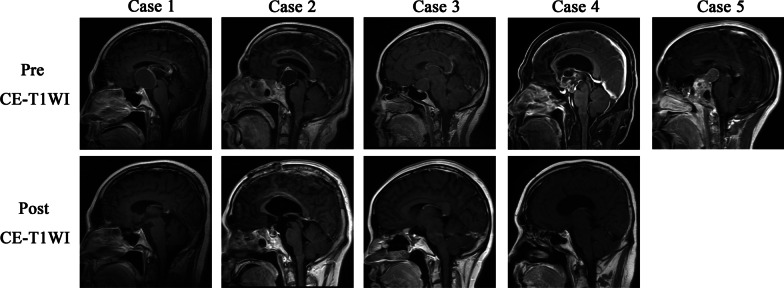
Pre- and postoperative magnetic resonance imaging of patients. The first line is the preoperative sagittal CE-T1WI MRI sequence. The second line is the sagittal CE-T1WI image obtained 3 months postoperatively, which is blank for patient 5 because the patient did not undergo surgery

**Fig. 2:**
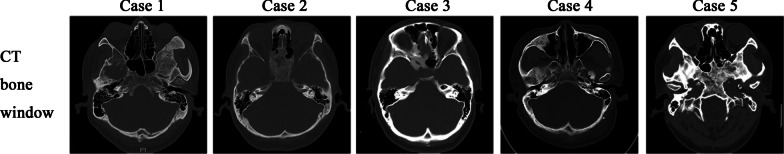
Preoperative computed tomography (CT) findings of the five patients. The patient's head CT bone window scan showed significant abnormal skull fibrous dysplasia

Table 2 summarizes the radiological characteristics of all five patients. Preoperative and 3-month follow-up MR images are shown in Fig. 1. The CPs were located in the sellar/suprasellar region (n = 5). Four tumors were suprasellar with extension to the third ventricle (patients 1–4), one tumor was purely suprasellar (patient 3), and two tumors had extension into the sellar region (patients 1 and 2). In contrast to CP in the general adult population, the CPs coexisting with CFD were cystic in our study (n = 5). The CPs of patients 1, and 2 showed hypointense on T1-weighted imaging (T1WI) sequence. The CPs of patient 3 showed isointensity on T1WI sequence. The CPs of patients 4, and 5 showed hyperintensity on T1WI sequence. All CPs in the five showed hyperintensity on T2-weighted imaging. After Gd-DTPA administration, the tumors showed inhomogeneous nodular and ring enhancement (n = 3) and ring enhancement (n = 2) on contrast-enhanced T1WI. Additionally, hydrocephalus was found in one patient (patient 4). The CFDs were located in the sphenoid bone (n = 4) and maxilla (n = 1), appearing as thickening of the skull bone with a mixed signal (n = 4). As shown in Fig. 2, CT showed typical CFD in all five patients, comprising mixed radiolucent manifestations in four and “ground-glass” manifestations in one. The sphenoid bone was most commonly affected by FD (n = 4), followed by the maxilla (n = 2), ethmoid (n = 1), and clivus (n = 1). And CT revealed calcifications in CPs of all five patients, including the cyst wall (n = 3), intrasellar region (n = 1), and within the tumor (n = 1).

**Table 2.** Radiological characteristics of all included 5 patients in our study

No.	CP							FD		
	Location	Pattern	size (cm)	MRI	CT	HP	CAL	Location	MRI	CT
Case.1	ITS, SPS, TV	Cystic	3.3 × 3.3 × 3.2	T1WI, HYPO; T2WI, HYPE; CET1WI, INRE	Low	No	Cyst wall	SW; Zygomatic process of maxilla (L)	Mixed; CET1WI, NE	Mixed
Case.2	ITS, SPS, TV	Cystic	2.4 × 2.6 × 3.7	T1WI, HYPO; T2WI, HYPE; CET1WI, INRE	Low	No	ITS	SD; Ethmoid	Mixed; CET1WI, NE	Mixed
Case.3	SPS	Cystic	2.0 × 2.2 × 2.2	T1WI, ISO; T2WI, HYPE; CET1WI, IRE	High	No	Cyst wall	SW (R)	T1WI, ISO; T2WI HYPO; CET1WI, NE	Ground-Glass
Case.4	SPS,TV	Cystic	3.3 × 2.8 × 4.0	T1WI, HYPE; T2WI, HYPE; CET1WI, INRE	Mixed	Yes	ITT	Maxilla (R)	Mixed; CET1WI, NE	Mixed
Case.5	SPS,TV	Cystic	1.7 × 2.0 × 2.2	T1WI, HYPE; T2WI, HYPE; CET1WI, IRE	Low	No	Cyst wall	SD; SW (L); Clivus,	Mixed; CET1WI, NE	Mixed

*CP* Craniopharyngiomas, *FD* Fibrous dysplasia, *MRI* Magnetic resonance imaging, *CT* Computed tomography, *HP* Hydrocephalus, *CAL* Calcification, *ITS* Intrasellar, *SPS* Suprasellar, *TV* third ventricle, *ITT* Intratumour, *SW* Sphenoid wing, *SD* Sphenoid body, *L* Left, *R* Right, *INRE* Inhomogeneous nodular and ring enhancement, *ISO* Isointensity, *IRE* Inhomogeneous ring enhancement, *HYPE* Hyperintensity, *HYPO* Hypointense, *NE* No enhancement, *T1WI* T1-weighted imaging, *T2WI* T2-weighted imaging, *CE-T1WI* contrast-enhanced T1-weighted imaging

### Endocrine abnormalities

Three patients had preoperative endocrinological deficits, including hypothyroidism (n = 2) and hypogonadism (n = 1). Patients 2 and 3 had hyperprolactinemia that was thought to be a result of stimulation of the pituitary stalk. Postoperative hormone deficits included panhypopituitarism (n = 2) and hypothyroidism (n = 2). Transient and permanent diabetes insipidus were each observed in one patient.

### Surgical approach and complications

Four of five patients underwent surgical resection of CPs (three transcranial and one trans-sphenoidal). Patient 5 refused surgical treatment because the lesion was found incidentally and there was no discomfort; the patient is still under close observation and regular follow-up. Patient 4 received a ventriculo-peritoneal shunt for hydrocephalus before tumor resection. Patient 3 underwent FD resection with optic nerve decompression; her vision was normal preoperatively, but was impaired postoperatively even after surgical repair. Extent of resection was confirmed both by intraoperative impression and immediate postoperative imaging in all cases. The extent of resection was considered gross total in two patients and subtotal in two patients.

Postoperative complications included panhypopituitarism (n = 2), diabetes insipidus (n = 2), visual loss (n = 1), hypothyroidism (n = 2), meningitis (n = 1), hypernatremia (n = 1), central fever (n = 1), and venous thrombus of the lower extremity (n = 1). No patient received any adjuvant therapy postoperatively.

### Histopathologic findings

**Fig. 3:**
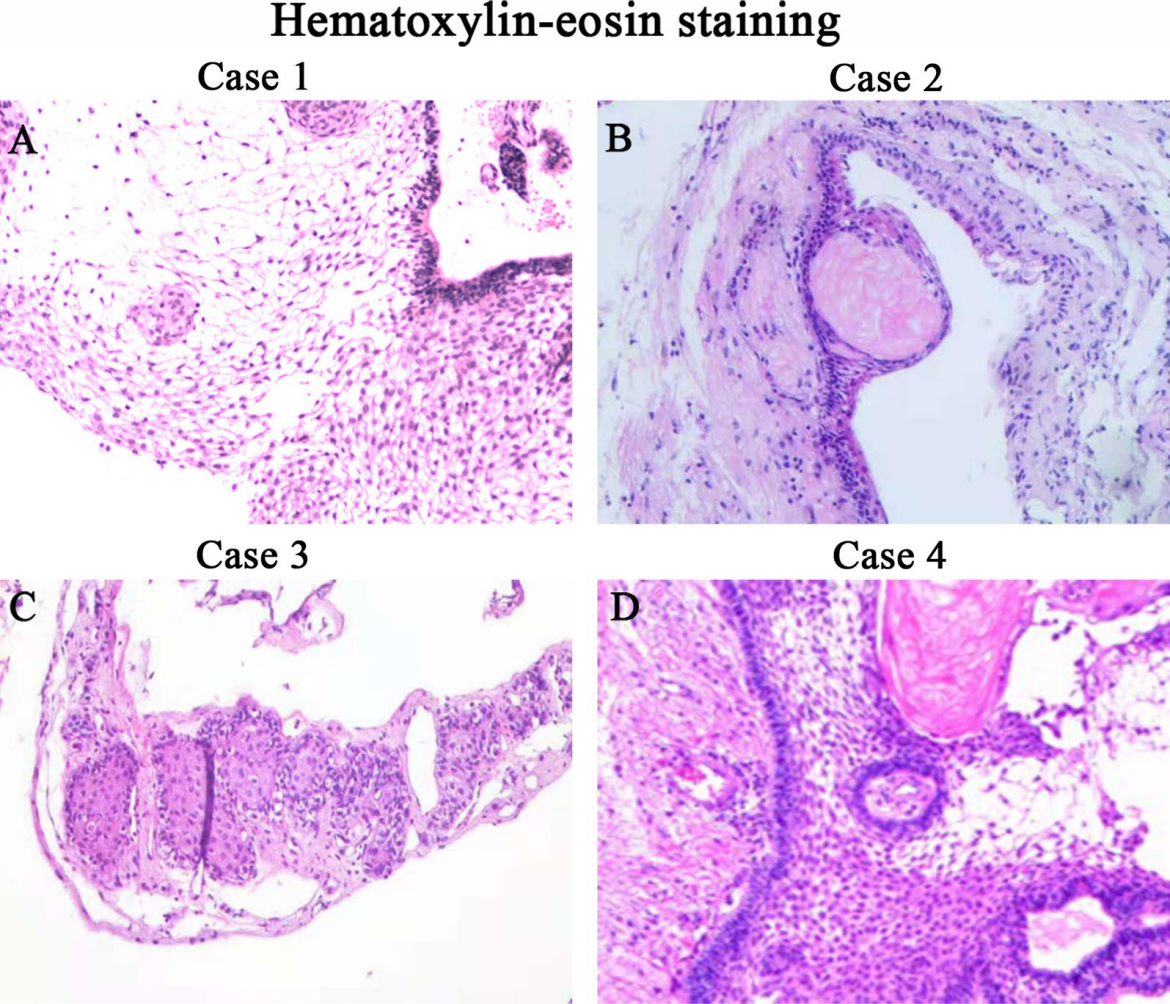
Pathological hematoxylin–eosin staining of the tumor specimens from patients 1–4. Three tumors were adamantinomatous craniopharyngiomas (ACPs) and one was a craniopharyngioma of an unknown subtype. **A** Histopathological examination reveals an ACP characterized by squamous epithelium arranged in a trabecular pattern as well as nodules of wet keratin in patient 1. **B** Postoperative pathology shows an ACP in patient 2. **C** Postoperative pathological examination shows a craniopharyngioma in patient 3; however, the specific subtype cannot be accurately identified. **D** An ACP that invaded and infiltrated the normal brain tissue in patient 4

**Fig. 4:**
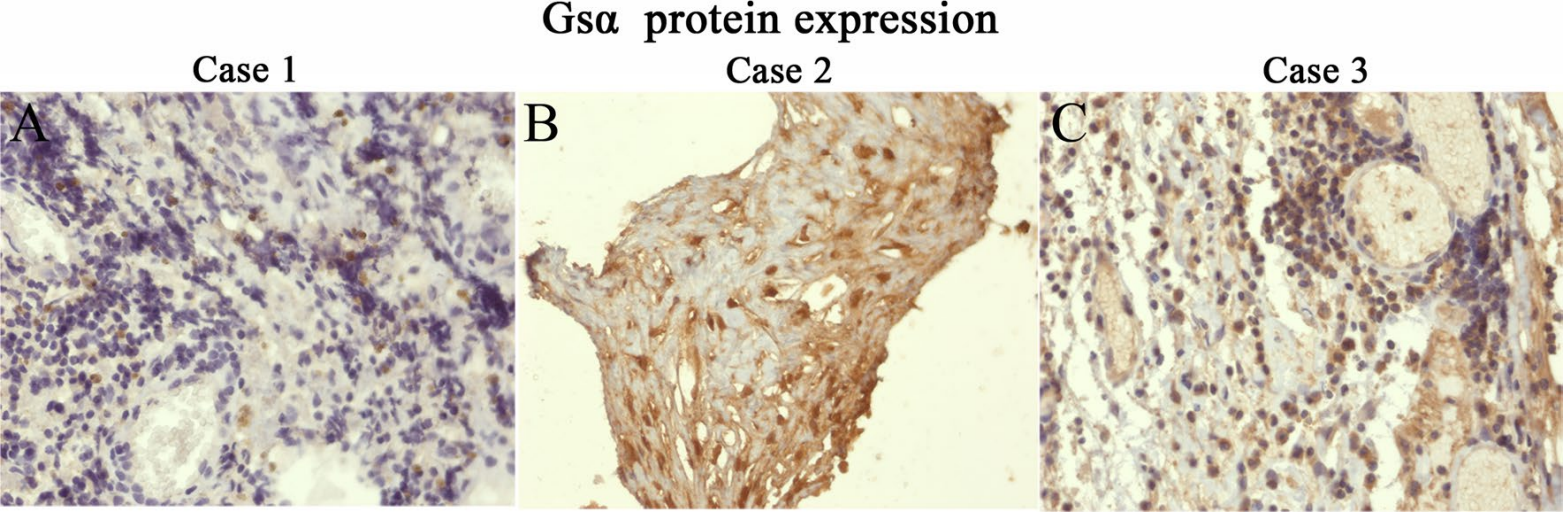
Immunohistochemical analysis of the Gsα expression of the craniopharyngiomas in patients 1–3. Gsα is strongly positively expressed in all three patients

The CP subtype was ACP in three patients and unidentified in one (Fig. 3). Patients 2 and 3 underwent DNA sequencing of the CP specimens, but no *GNAS* mutations were detected (Additional file 1: Figure S1). However, immunohistochemical examinations revealed a strong positive Gsα expression (Fig. 4).

### Follow-up and outcome

The median follow-up period was 57.2 months (range, 6–98 months). During the follow-up period, one patient (patient 1) experienced radiological recurrence and received long-term hormone replacement therapy for permanent panhypopituitarism. The CP in patient 4 was obviously calcified, closely related to blood vessels, and pathologically showed brain invasion (Additional file 2: Figure S2), making the resection very difficult. Postoperatively, the hypothalamic/pituitary function of patient 4 was severely damaged, causing water and electrolyte imbalance, lower extremity venous thrombosis, and eventually death from pulmonary embolism caused by thrombosis at 6 months postoperatively.

## Discussion

To the best of our knowledge, this is the first reported case series of concomitant CP and CFD that highlights the diagnostic and management challenges. In addition to summarizing the clinicopathologic features, treatment modalities, and outcomes of these patients, we also reviewed the literature and discussed possible links between these two conditions based on genetic mutation detection and immunohistochemical studies.

The incidence of the coexistence of primary brain tumors of different pathologies is low and the most common association is meningioma and glioma, followed by meningioma and pituitary tumor, as these are the most common primary intracranial tumors [9]. The definitive pathogenesis of such coexistence of primary brain tumors remains unknown, except in patients with familial or hereditary diseases such as neurofibromatosis type 2 or multiple endocrine neoplasia type 1. Controversial hypotheses that have been advocated to explain the coexistence of primary brain tumors of different pathologies include pure coincidence, surgical trauma, radiation and/or chemical exposure, and genetic predisposition [10–12].

Because the sellar region undergoes a complex process of embryonic development, it is easy for primary intracranial tumors of different cell types to coexist in this region at the same time. However, it is extremely rare for CPs to coexist with other central nervous system neoplasms. To the best of our knowledge, there have only been a few reported cases of CPs coexisting with other central nervous system tumors, and the most common concomitant tumors are pituitary adenomas and meningiomas. The latest review reported that only 22 patients with CP (median age 47.0 years; 15 men, seven women) had an associated pituitary adenoma, and the CP was an ACP in 19 of these 22 patients [13]. Liu et al. reported eight patients (median age 62.0 years; five men, three women) with concomitant CP and meningioma, and five of these eight patients had ACPs [14]. Similarly, in our series (median age 39 years; five men, one woman), three of four operative patients had ACPs. These findings suggest that such coexistence is more likely to occur in adults with ACPs, with an obvious male predominance. In contrast, ACP that occurs alone has no sex orientation and is more common in adolescents [15]. Therefore, there may be some underlying reason for the coexistence of CPs with other central nervous system diseases rather than pure chance, and some factors may stimulate ACP cells to induce a secondary tumor formation, which is quite different from papillary CPs.

FD can also occur in association with other primary brain tumors under two conditions. The first is purely accidental and involves the coexistence of FD with the most common brain tumor (meningioma), which has been reported in only a few cases [16, 17]. The second is the coexistence of FD with pituitary adenoma (possibly in accordance with MAS), which has been reported in dozens of cases. As a postzygotic disease, the clinical spectrum and severity of FD varies depending on the timing of the mutation [18]. In addition to the classic triad, MAS may have a broad spectrum or atypical presentations [19]. Furthermore, MAS may be associated with gastrointestinal disease and breast cancer [20, 21]. Although no *GNAS* mutations were detected in the CP specimens in our series, we should not completely exclude the possibility of an atypical FD/MAS course, as immunohistochemical staining showed a high expression of Gsα protein in the three CP specimens. Further studies are needed to clarify this issue.

Most CPs or CFDs are diagnosed correctly based on the typical clinical symptoms and imaging features. However, it is difficult to accurately and promptly diagnose the coexistence of CP and CFD before surgery because the imaging features of FDs and CPs are variable and mutually influenced [22, 23]. Deformation and thickening of the skull base might make the situation more confusing, especially when the two lesions are adjacent to each other. However, it is important when devising a treatment strategy preoperatively to confirm which lesion is responsible for the clinical symptoms. In our case series, the four patients who underwent surgical treatment were accurately diagnosed preoperatively.

There are currently no established treatment strategies for concomitant CP and CFD. The current standard treatment for CP is surgical resection via a transcranial or extended endonasal endoscopic approach, followed by adjuvant radiation therapy if required for residual tumor [24]. Given the benign nature of CFD and the fact that the patients are adults, observation is considered adequate for asymptomatic quiescent FD [25]. However, all the primary symptoms (such as headache, fatigue, polydipsia/polyuria, and visual impairment) of all patients in our study were considered to be caused by CPs; thus, our management strategy was to surgically resect the CP and regularly monitor the FD. With the progress of microsurgery and endoscope technology, the surgical obstacles and risks caused by FD-related skull hyperplasia and deformation will be gradually reduced. Our results also show that there were no complications directly related to surgery, except for vision deterioration in patient 3 because of preventive decompression of the optic canal encased by FD. It should also be noted that FD radiotherapy may produce adverse effects of malignant transformation [26].

The outcome of collision tumors composed of CP and CFD are undefined because of the small number of reported cases. In our experience, the outcome of such tumors is similar to that of sporadic CPs, and long-term morbidity is associated with tumor- and treatment-related risk factors.

Our study has some limitations. First, due to the extreme rarity of concomitant CP and CFD, the total number of cases was small; more cases are needed to strengthen the reliability of data. Second, the mechanism of the coexistence of CP and CFD still needs further exploration and verification; technologies such as whole exome sequencing can be used to study the mechanism of the coexistent relationship between CP and CFD in future research.

## Conclusion

We reported a very rare case series of concomitant CP and CFD and provided a detailed description of the clinicopathological features, treatment, and outcome. A comprehensive assessment of this rare and complicated situation is warranted to determine the appropriate treatment strategy. The clinical course of our cases show that an individual treatment plan for CP or CFD is required and can lead to a good outcome. The pathogenesis of the coexistence of the two tumor types is still undefined and requires investigation in further studies.

## Supplementary Information

**Additional file 1: Figure S1.** DNA sequencing of GNAS in the craniopharyngiomas of patients 2 and 3. The mutations in codon 227 and 201 of GNAS are characteristic of fibrous dysplasia. No mutations were found in the craniopharyngioma samples of patients 2 and 3. The specimen from patient 1 could not be adequately assessed because the craniopharyngioma had little cystic wall tissue.

**Additional file 2: Figure S2.** Head computed tomographic angiography of patient 4 shows that the tumor is closely associated with the blood vessels, increasing the difficulty of the operation

## Data Availability

The datasets generated and/or analysed during the current study are not publicly available due to individual privacy of the patients included but are available from the corresponding author on reasonable request.
