# Supplementary figures and images for: Coexistence of craniopharyngioma and cranial fibrous dysplasia: a case series of clinicopathological study
Source: Orphanet J Rare Dis. 2022 Mar 18;17:126. doi: 10.1186/s13023-022-02281-1 (PMC8932149; doi:10.1186/s13023-022-02281-1)

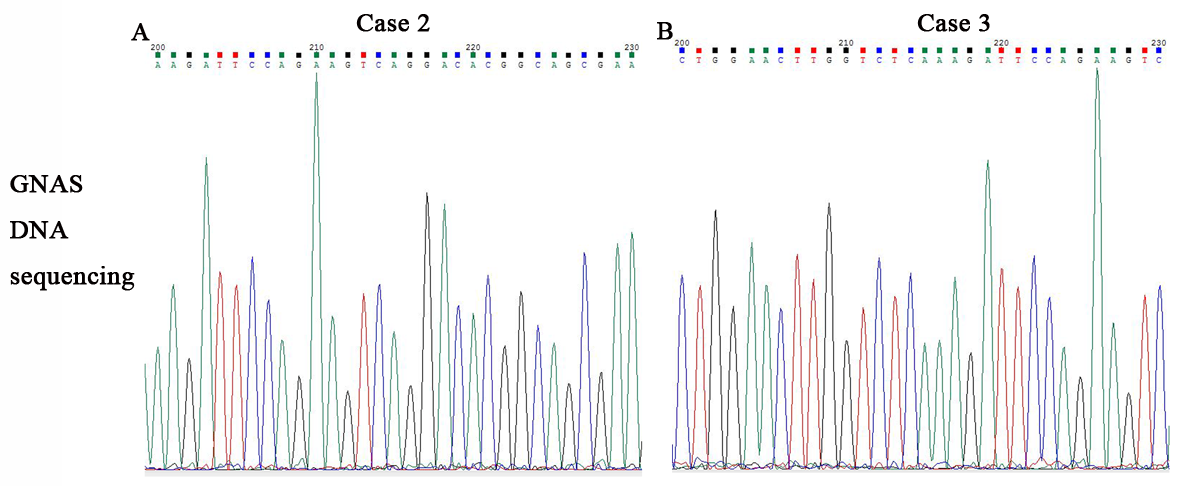

Supplement: Supplementary file 1 — Additional file 1: Figure S1. DNA sequencing of GNAS in the craniopharyngiomas of patients 2 and 3. The mutations in codon 227 and 201 of GNAS are characteristic of fibrous dysplasia. No mutations were found in the craniopharyngioma samples of patients 2 and 3. The specimen from patient 1 could not be adequately assessed because the craniopharyngioma had little cystic wall tissue. [file 13023_2022_2281_MOESM1_ESM.tif]

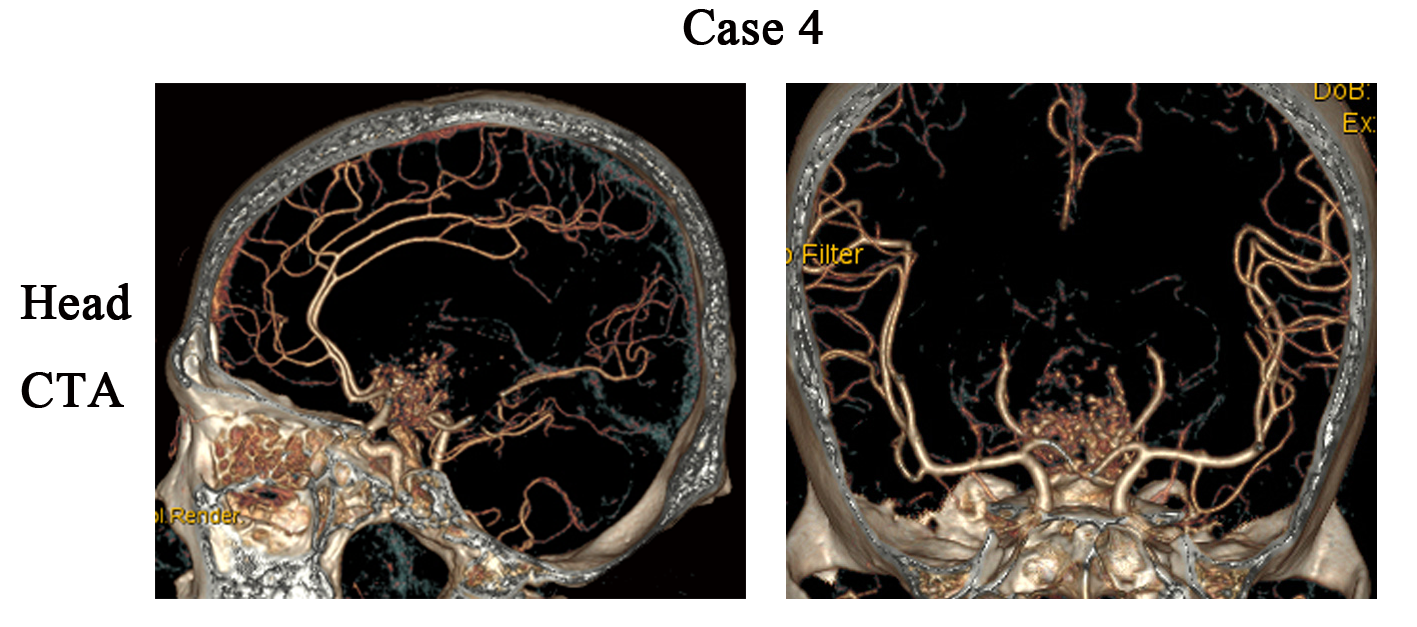

Supplement: Supplementary file 2 — Additional file 2: Figure S2. Head computed tomographic angiography of patient 4 shows that the tumor is closely associated with the blood vessels, increasing the difficulty of the operation [file 13023_2022_2281_MOESM2_ESM.tif]
